# Daily Streamflow of Argentine Rivers Analysis Using Information Theory Quantifiers

**DOI:** 10.3390/e26010056

**Published:** 2024-01-09

**Authors:** Micaela Suriano, Leonidas Facundo Caram, Osvaldo Anibal Rosso

**Affiliations:** 1 Departamento de Hidráulica, Facultad de Ingeniería, Universidad de Buenos Aires, Av. Las Heras 2214, Buenos Aires C1127AAR, Argentina; 2 Laboratorio de Redes y Sistemas Móviles, Departamento de Electrónica, Facultad de Ingeniería, Universidad de Buenos Aires, Buenos Aires C1063ACV, Argentina; fcaram@fi.uba.ar; 3 Instituto de Física (IFLP), Universidad Nacional de La Plata, CONICET, La Plata B1900AJJ, Argentina; oarosso@gmail.com; 4 Instituto de Física, Universidade Federal de Alagoas (UFAL), Maceió 57072-970, Brazil

**Keywords:** permutation entropy, statistical complexity, streamflow series, Argentine rivers

## Abstract

This paper analyzes the temporal evolution of streamflow for different rivers in Argentina based on information quantifiers such as statistical complexity and permutation entropy. The main objective is to identify key details of the dynamics of the analyzed time series to differentiate the degrees of randomness and chaos. The permutation entropy is used with the probability distribution of ordinal patterns and the Jensen–Shannon divergence to calculate the disequilibrium and the statistical complexity. Daily streamflow series at different river stations were analyzed to classify the different hydrological systems. The complexity-entropy causality plane (CECP) and the representation of the Shannon entropy and Fisher information measure (FIM) show that the daily discharge series could be approximately represented with Gaussian noise, but the variances highlight the difficulty of modeling a series of natural phenomena. An analysis of stations downstream from the Yacyretá dam shows that the operation affects the randomness of the daily discharge series at hydrometric stations near the dam. When the station is further downstream, however, this effect is attenuated. Furthermore, the size of the basin plays a relevant role in modulating the process. Large catchments have smaller values for entropy, and the signal is less noisy due to integration over larger time scales. In contrast, small and mountainous basins present a rapid response that influences the behavior of daily discharge while presenting a higher entropy and lower complexity. The results obtained in the present study characterize the behavior of the daily discharge series in Argentine rivers and provide key information for hydrological modeling.

## 1. Introduction

Proper water management has always been a key to societies’ progress. For this reason, solutions have been developed to modify spatial and temporal water availability to adapt it to human needs. In the global context of climate change that affects the region, it is imperative to assess present and future water availability to optimize water resource management and planning.

The hydrological cycle is a complex system [1]. It consists of a coordinated and balanced interaction between the atmosphere, the ocean, and the land that controls the planet’s temperature. It moves a large amount of matter and energy, which results in a highly variable process in space and time, and this variability exists at all scales, from centimeters to the continent scale, from minutes to years [2]. For these reasons, hydrological modeling plays a crucial role in capturing the complex hydrology of large basins and informing basin development decisions through comprehensive modeling approaches [3].

A classification framework that categorizes basins into different groups and sub-groups is necessary for more effective and efficient model selection and generalization in the modeling approach [4,5,6]. For example, catchment classification can be used in transboundary rivers that face water usage competition across distinct hydrological characteristics [7]. The information can aid stakeholders in irrigation planning, navigation, and flood risk management, among others.

A framework is required to classify basins into different groups based on their hydrological characteristics. Nonlinear dynamic concepts offer a suitable methodology. Several studies indicate that complexity plays a part in the classification of hydrological systems [8,9,10].

For this purpose, the complexity-entropy causality plane (CECP) has been introduced as a diagnostic diagram. It plots statistical complexity versus entropy, applying nonlinear dynamics analysis to classify signals according to their degrees of randomness and complexity [11,12]. This methodology presents the temporal relationship among the values of the time series, taking into account time causality [13].

There are several applications in the field of hydrology. Almost 500 series of daily discharge rivers from different countries were studied in [14]. The results show that the data are a mixture of random and deterministic processes. A comparison with the k-noise series in the CECP is proposed to quantify the observed differences. In [15], the CECP was conducted for the analysis of 80 series of daily flows at different stations in the United States over a period of 75 years. It was found that both chaotic and stochastic systems can be compatible with the daily streamflow dynamics and that the CECP can differentiate the signals in the presence of moderate observational noise.

Another application of the methodology is to analyze the behavior in the flow series relating to changes in the basin, such as the construction of dams or land use change, as well as the influence of long-term climatic phenomena. An example of this application is presented in [16,17], which shows the influence of the construction of the Sobradinho dam on the series of daily flows of the São Francisco River in Brazil. The study found different patterns of complexity and entropy. In addition, the close relationship between the flow dynamics and the El Niño Southern Oscillation phenomenon was confirmed.

In [18], statistical complexity was used as a metric for hydrological alteration at the basin scale. The daily streamflow records of 22 urban watersheds in US cities were studied to analyze hydrological changes due to urbanization. The findings indicate that, in urban watersheds, there is a tendency for a decrease in complexity and an increase in entropy as hydrological alteration intensifies.

This paper analyzes the temporal evolution of stream flows in different rivers in Argentina, utilizing information quantifiers to identify the complexity-entropy causality plane, which consequently allows for the classification of the different hydrological systems. Argentina is a large country; the continental area is 2,791,810 km^2^ (Instituto Nacional de Estadística y Censos de la República Argentina (INDEC)) and water resources are not uniformly distributed. There are basins of different sizes with varying characteristics depending on their location. Also, a high percentage of the territory is characterized by arid or semi-arid climates, where water demand exceeds availability.

This study aims to improve knowledge about the behavior of the daily discharge series selected, providing relevant information for hydrological modeling. This work is organized into the following sections: Methodology: description of applied methods and equations, Data: description of the source of information and characteristics of the used time series, Results: development of the outcomes, interpretation analysis, and Conclusions: a summary of the main conclusions obtained from this work.

## 2. Materials and Methods

### 2.1. Methodology

For a given arbitrary probability distribution ***P*** = {*p_i_*: *i* = 1, …, *N*}, the Shannon logarithmic information measure is defined by:

(1) $$S\left[P\right]=-\sum_{i=1}^{N}p_{i}\ln\left(p_{i}\right)\;,$$

and it is considered a measure of the uncertainty associated with the physical processes described by ***P***. If $S\left[P\right]$ = 0, it is possible to predict with certainty which of the possible scenarios i, with associated probabilities given by *p_i_*, will actually occur. On the contrary, our ignorance is at its maximum for a uniform distribution. In [19], it is presented as a measure of statistical complexity capable of detecting key details of the dynamics. This is defined through the product:

(2) $$C_{JS}\left[P\right]=Q_{J}\left[P,P_{e}\right]{\;.\;H}_{S}\left[P\right],$$

and the generalized Shannon entropy [20] is:

(3) $$H_{S}=\frac{S\left[P\right]}{S_{máx}}$$

with *S_máx_* = *S*[***P_e_***] *= ln*(*N*), 0 ≤ *H*_s_ ≤ 1 and ***P_e_*** *=* 1/*N*, *…*, 1/*N* the uniform distribution.

The disequilibrium is defined in terms of the Jensen–Shannon divergence:

(4) $$Q_{j}\left[P,P_{e}\right]=Q_{0}\;J\left[P,P_{e}\right],\;\;\;\;$$

being *Q*_0_ a normalization constant equal to the inverse of the maximum possible value of *J*[***P***,***P_e_***] and the Jensen–Shannon divergence:

(5) $$J\left[P,P_{e}\right]=S\left[\frac{P+P_{e}}{2}\right]-\frac{S\left[P\right]}{2}-\frac{S\left[P_{e}\right]}{2}$$

The method of ordinal patterns [13], developed by Bandt and Pompe (2002), is applied to determine the probability distribution function ***P***, as it takes into account the temporal causality within the dynamics of the process.

The approach is based on the sequence of values that occurs in the time series, which is replaced by the corresponding range sequence. Given a time series {*x_t_*: *t* = 1, …, *N*}, an embedding dimension *D* ≥ 2 (*D* ∈ ℕ), and the delay time *τ* (*τ* ∈ ℕ), the D-ordinal pattern is generated by:

(6) $$s\to \left\{X_{s-\left(D-1\right)\tau },{\;X}_{s-\left(D-2\right)\tau },\;\ldots ,\;X_{s-\tau },\;X_{s}\right\}$$

For every time instant *s*, a *D*-dimensional vector is assigned, and it results from the evaluation of the time series in the *s* − (*D* − 1) *τ*, *…*, *s − τ*, *s* instants. A greater *D* value means greater information about the pass incorporated in the resultant vector. The *D*-ordinal patterns related to the instant *s* are referred to as the permutation ***π*** = {*r*_0_, *r*_1_, *…*, *r_D−_*_1_} of {0, 1, …, *D −* 1} defined by:

(7) $$x_{s-r_{0}\tau }\geq x_{s-r_{1}\tau }\geq \cdots \geq x_{s-r_{D}-2\tau }\geq x_{s-r_{D}-1\tau }.\;\;$$

**Figure 1:**
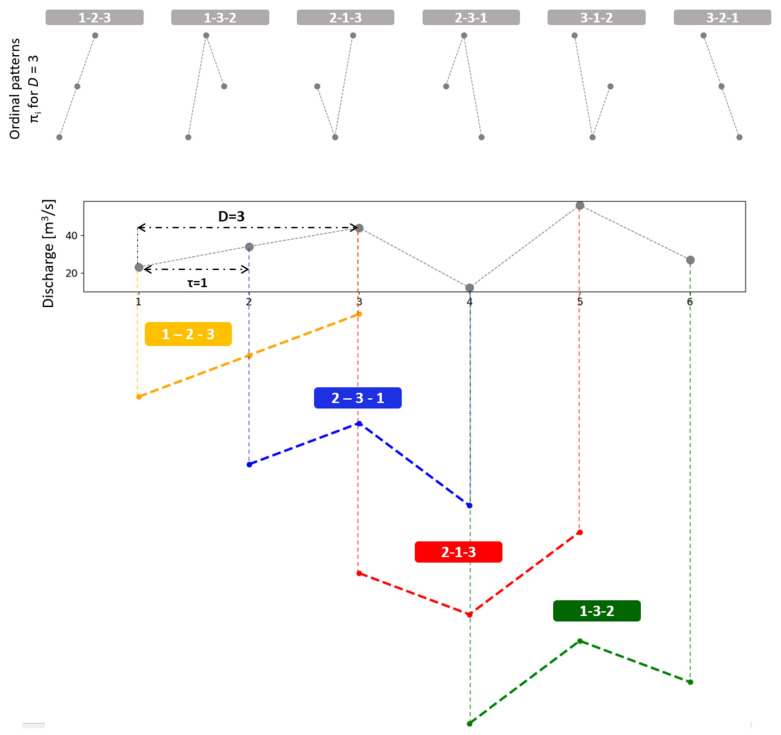
An example to illustrate the ordinal patterns methodology [13] applied to a discharge time series for an embedding dimension *D* = 3 and an embedding delay *τ* = 1. The possible combinations (ordinal patterns *π*_i_) for *D* = 3 are presented at the top. The analysis for a discharge series with *τ* = 1 is found at the bottom. By replacing the original values with their corresponding rankings, the pattern is obtained (adapted from [21]).

In this way, the vector defined by Equation (6) becomes the unique symbol **π**. With the objective of finding a unique result, $r_{i}<r_{i-1}\;if\;x_{s-r_{i}\tau }<x_{s-r_{i-1}\tau }$ is considered. As the value of *x_t_* has a continuous distribution, equal consecutive values are unusual. A graphic example of the determination of the permutations π_i_ from order *D* = 3 and *τ* = 1 is shown in Figure 1. The possible combinations (ordinal patterns **π_i_**) for *D* = 3 are presented at the top of Figure 1, while the analysis for a discharge series and *τ* = 1 is found at the bottom. For every possible *D!* order (permutations) **π_i_** from order *D*, their associated relative frequencies can be computed as the number of times that this sequence appears in the series, and thus the number divided by the total of sequences. Consequently, the ordinal pattern distribution probability for the time series given is obtained.

Regarding the selection of the parameters, Bandt and Pompe [13] applied 3 ≤ *D* ≤ 7 and *τ* = 1. The specific value of *D* should be the minimum sampling frequency that retains all the information about the time structure of the signal [15]. To distinguish between deterministic and stochastic dynamics, it is suggested that *N* ≫ *D*!, where *N* represents the length of the time series [12]. The selection of *τ* = 1 captured the daily temporal structure of the discharge series [18], but long-term temporal variabilities were studied in [15].

**Figure 2:**
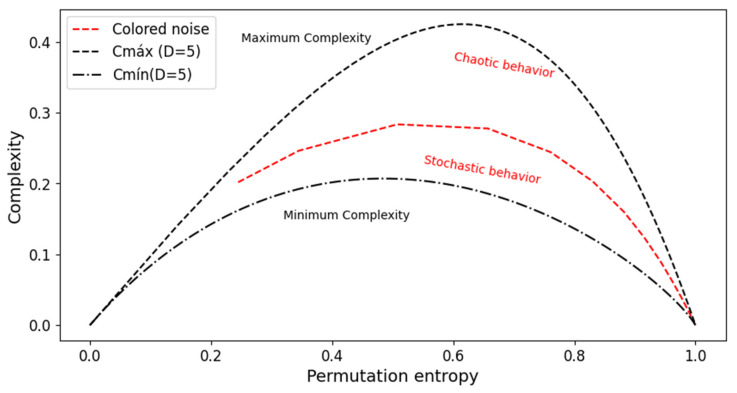
Schematic representation of the complexity-entropy causality plane (CECP) based on [22]. Maximum and minimum boundaries for *D* = 5 are represented, as well as colored noise with the approximate representation of chaotic and stochastic zones.

The complexity-entropy causality plane (CECP) is the representation of plotting the permutation statistical complexity *C_JS_* versus the generalized Shannon entropy *H_S_* and the bounds for an admissible region that only depends on the embedding dimension *D* [8,9]. The complexity remains within the bounds of minimum and maximum complexity, and a maximum and minimum envelope complexity as a function of the entropy can be calculated [22]. A schematic illustration of the CECP is shown in Figure 2. Maximum and minimum boundaries are calculated for *D* = 5 (Cmáx and Cmín, respectively) with the approximate representation of chaotic and stochastic zone classification.

Another graphic representation is the Fisher–Shannon plane, which is calculated using Bandt and Pompe [13] to determine the probability distribution of a time series. This method can uncover the informational properties of the planar location [23]. The Fischer–Shannon casualty plane *H_s_ x F* is calculated, where *H_s_* is the generalized Shannon entropy in Equation (3) and *F* is the Fisher’s information measure (FIM) [24] as a measure of the gradient content of the distribution *f*(*x*), as follows:

(8) $$F\left[f\right]=\int_{∆}\frac{1}{f(x)}{\left[\frac{df(x)}{dx}\right]}^{2}dx=4\int_{∆}{\left[\frac{d\Psi (x)}{dx}\right]}^{2}.\;\;$$

FIM could be interpreted as a measure of the ability to estimate the amount of information that can be extracted from a set of measurements [25]. For a discrete environment, the best-behaved expression to use [26] is the discrete normalized FIM, as given by:

(9) $$F\left[P\right]=F_{0}\sum_{i=1}^{N-1}{\left[{\left(p_{i+1}\right)}^{\frac{1}{2}}-{\left(p_{i}\right)}^{\frac{1}{2}}\right]}^{2}\;\;$$

and the normalization constant *F*_0_ is given by:

(10) $$F_{0}=\left\{\begin{array}{c} 1\;if\;p_{i*}=1\;for\;i^{*}=1\;or\;i^{*}=N\;and\;p_{i}=0\;\forall i\neq i^{*}\\ 0\;otherwise\; \end{array}\right\}\;\;\;$$

The algorithms are obtained from the Python library *ordpy*: A Python package for data analysis with permutation entropy and ordinal network methods [27]. The color noise series was generated with the library *colorednoise* (by Felix Patzel on github) that generates Gaussian distributed noise with a power law spectrum based on the algorithm in [28]. Plots are created using the *matplotlib* [29], *seaborn* [30], and *geopandas* [31] Python libraries.

To summarize, the main statistical tools used in this paper are as follows:

- Permutation entropy is a measure of the uncertainty associated with the analyzed process and is calculated by generalized Shannon entropy [20] and ordinal patterns [13].
- Statistical complexity is a measure capable of detecting key details of the dynamics [19].
- Ordinal patterns are a methodology to determine the probability distribution function of the analyzed time series. It takes into account the temporal causality within the dynamics of the process [13].
- The complexity-entropy causality plane is a graphical representation for visualizing and classifying the behavior of time series. Obtained by plotting values of statistical complexity against permutation entropy [8,9,17].
- A Fisher information measure is a representation of the ability to estimate the amount of information that can be extracted from a time series. The Fisher–Shannon representation plane is another visualization tool for characterized time series [24,25].

### 2.2. Data

The methodology was applied to 14 daily discharge series at different hydrometric stations across Argentina. The data were obtained from the National Hydrological Network of the National Water Information System of the Secretariat of Infrastructure and Water Policy (Sistema Nacional de Información Hídrica: https://www.argentina.gob.ar/obras-publicas/hidricas/base-de-datos-hidrologica-integrada, accessed on 19 February 2021).

**Figure 3:**
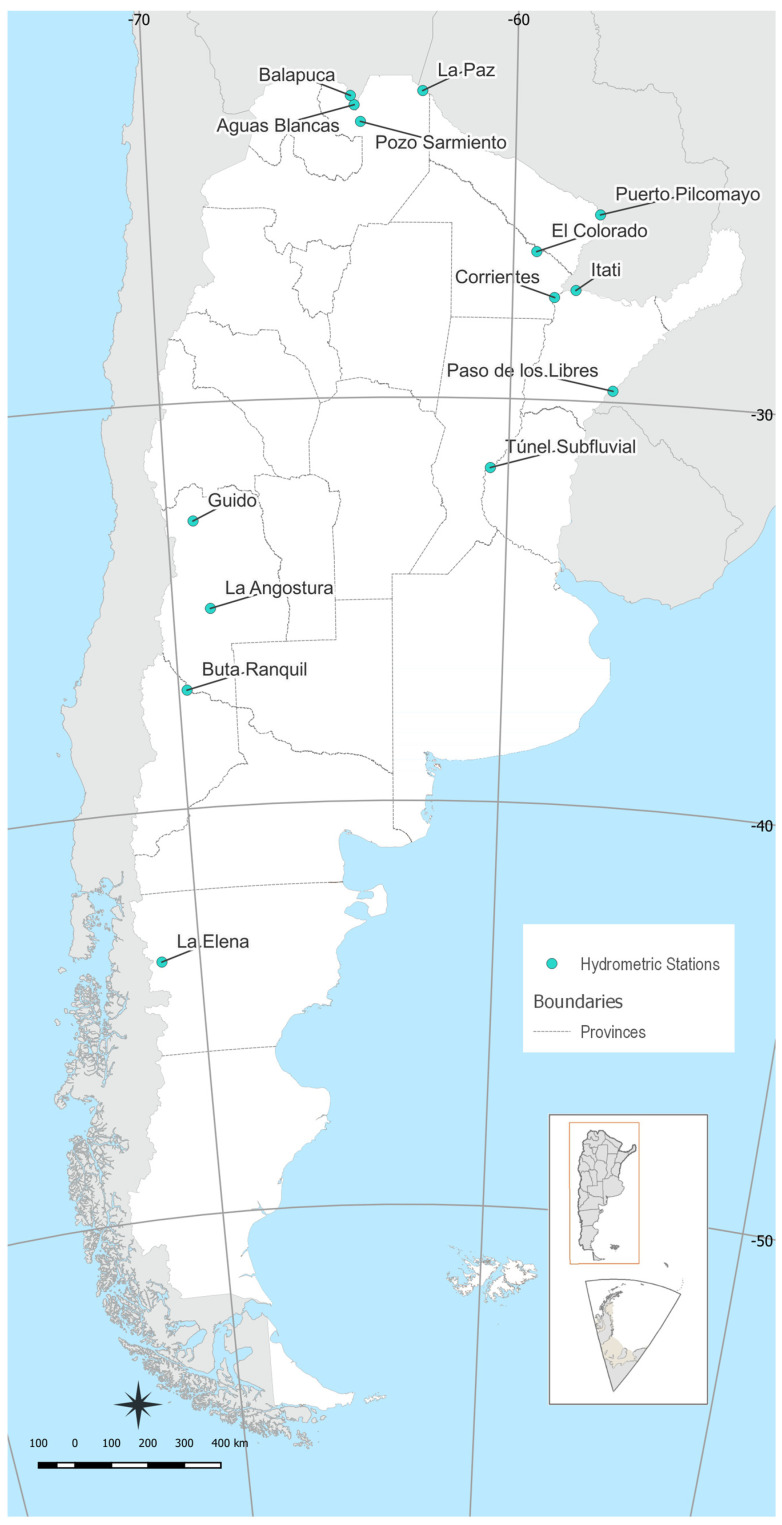
Geographical locations of the 14 hydrometric stations assessed in this study for analyzing the daily discharge series. The map displays the extent of Argentina along with its provincial and international boundaries (adapted from: the Instituto Geográfico Nacional de la República Argentina).

The main information associated with the stations is shown in Table 1, and their geographical locations are presented in Figure 3. The selection of data was based on the length and completeness of the discharge series (almost more than 50 years of record) and on its ability to represent different types of hydrological processes. Generally, the selected stations in the southwest of the country are located in smaller basins, and the streamflow is mainly produced by snow, while the discharge in the northwest of Argentina is generated by rainwater.

The daily discharge series from each hydrometric station was downloaded, followed by an analysis of the missing data. Among the 14 series examined, 11 exhibited less than 3% missing data, one series had less than 5%, and two series had less than 10%. Subsequently, a daily deseasonalized discharge series was calculated for each daily discharge series with the objective of removing seasonal patterns. The daily deseasonalized discharge series was obtained from the equation:

(11) $${Qd}_{i,j,k}=\frac{Q_{i,j,k}-{\bar{Q}}_{i,j}}{\sigma_{i,j}},$$

where ***Q***_*di*,*j*,*k*_ is the discharge deseasonalized for the *i-th* day, *j-th* month, and *k-th* year; ***Q****_i_*_,*j*,*k*_ is the daily discharge for the *i-th* day, *j-th* month, and *k-th* year; ${\bar{Q}}_{i,j}$ is the average of all the *i* days and *j* month; and $\sigma_{i,j}$ is the standard deviation of all *i* days and *j* month for every year of the record [32].

**Figure 4:**
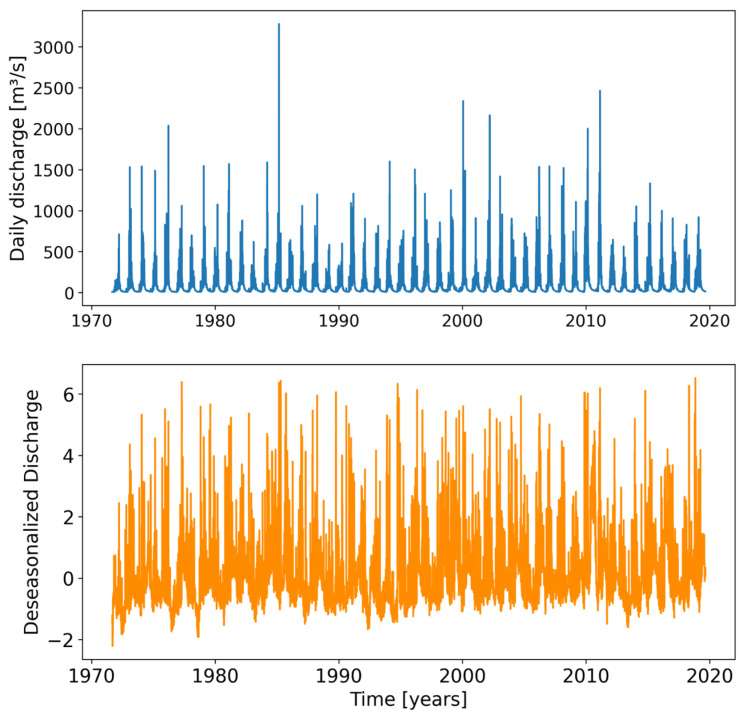
Daily discharge (**above**) and deseasonalized discharge (**bottom**) series for the Bermejo River at Balapuca hydrometric station (data from https://www.argentina.gob.ar/obras-publicas/hidricas/base-de-datos-hidrologica-integrada, accessed on 19 February 2021).

An example of daily discharge and deseasonalized discharge series for the Bermejo River at Balapuca station is shown in Figure 4. The same procedure was applied to all series of the stations listed in Table 1.

## 3. Results and Discussion

**Figure A1:**
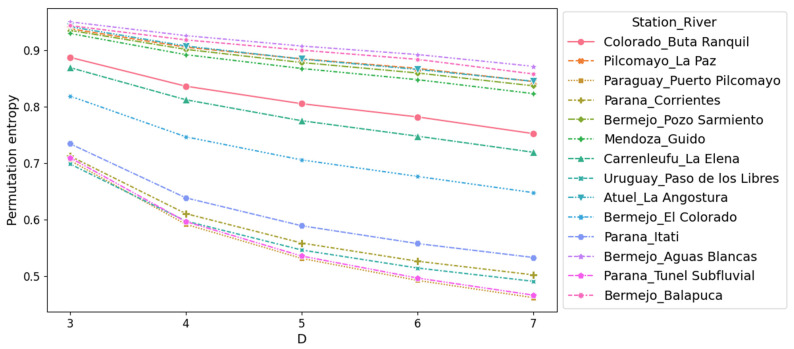
Permutation entropy for daily deseasonalized discharge series, varying parameter *D*.

**Figure A2:**
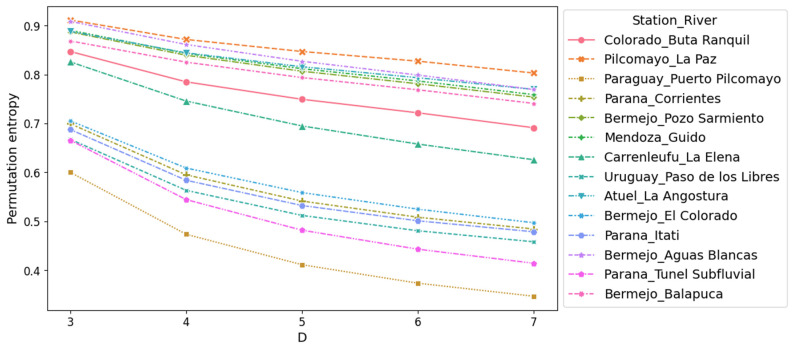
Permutation entropy for daily discharge series, varying parameter *D*.

**Figure A3:**
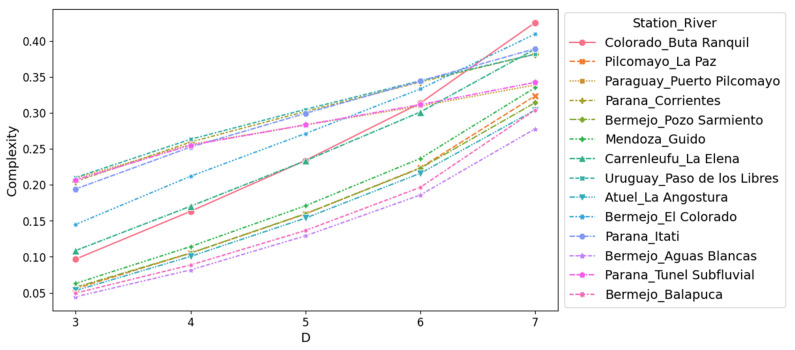
Complexity for daily deseasonalized discharge series, varying parameter *D*.

**Figure A4:**
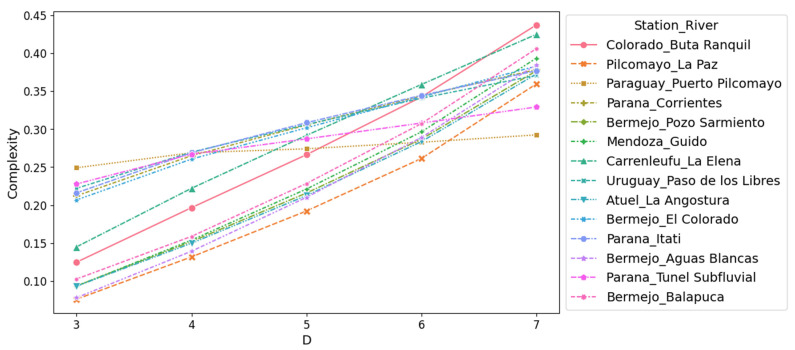
Complexity for daily discharge series, varying parameter *D*.

The results obtained from applying the methodology to the daily discharge and the deseasonalized discharge series are shown in Appendix A. The permutation entropy and statistical complexity were calculated for *τ* = 1 and varying *D* from 3 to 7 (Figure A1, Figure A2, Figure A3 and Figure A4).

The permutation entropy and complexity were applied to the daily discharge and deseasonalized discharge series for 3 < *D* < 7. In both cases, the permutation entropy decreases while the parameter *D* increases. For the complexity measure, the behavior is different. The complexity and the parameter *D* increase simultaneously, but the slope is different among the time series studied. A possible explanation could be related to the fact that the record period for the different series is not the same.

**Figure A5:**
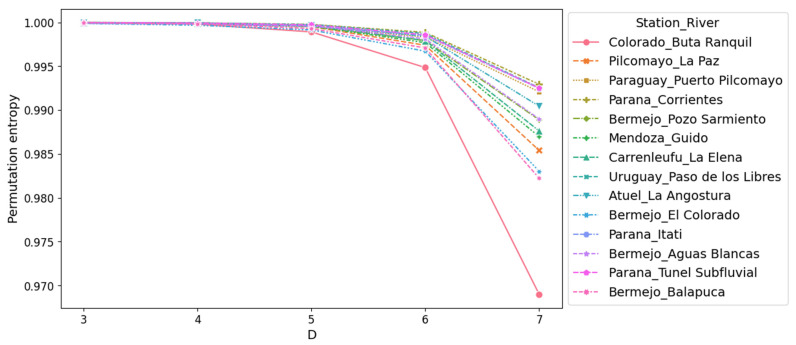
Permutation entropy for a shuffled series of the daily discharges, varying parameter *D*.

**Figure A6:**
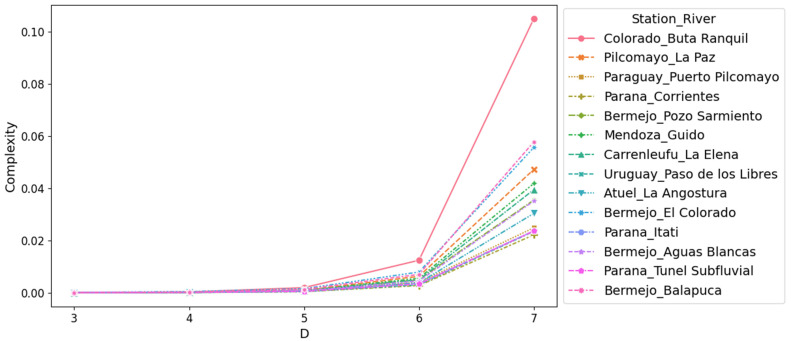
Complexity for a shuffled series of the daily discharges, varying parameter *D*.

**Figure A7:**
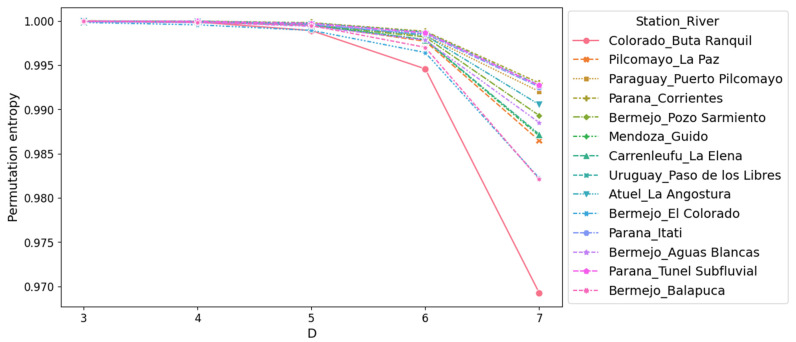
Permutation entropy for a shuffled series of the daily deseasonalized discharge series, varying parameter *D*.

**Figure A8:**
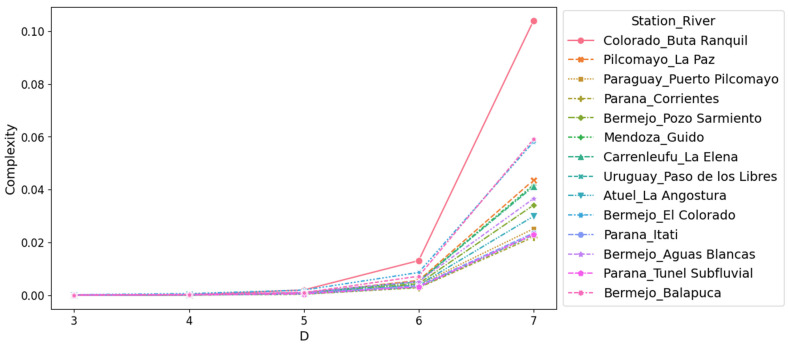
Complexity for the shuffled series of the daily deseasonalized discharge series varying parameter *D*.

The same analysis was performed for the shuffled series of daily discharge and deseasonalized discharge series (Appendix A: Figure A5, Figure A6, Figure A7 and Figure A8). The results showed that in both cases, the entropy approaches 1 and the complexity approaches 0 for daily discharge and for deseasonalized discharge time series. These results indicated that the sorting of the values has an impact on the behavior of the time series in terms of entropy and complexity. These results showed that, for the studied discharge time series, the sequential arrangement of values significantly influences the process structure and the information obtained from it.

For future analysis in this study, *D* = 5 will be considered, following the criteria that the record length of the series *N* should be *N* >> *D*!.

**Figure 5:**
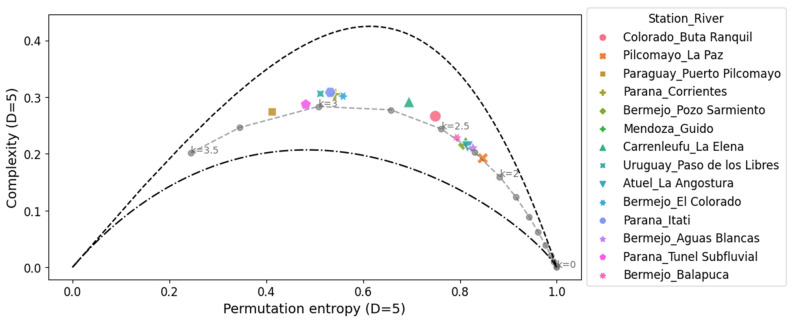
Complexity-entropy causality plane for all the daily discharge series analyzed and noise with power spectrum frequency-varying parameter *k* (gray dashed line). The parameters used are *D* = 5 and τ = 1.

**Figure 6:**
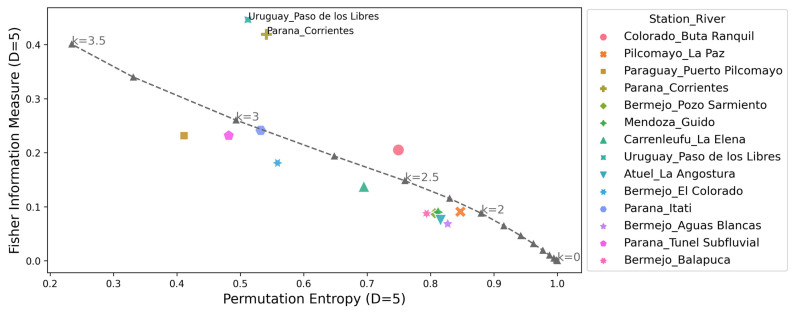
Fisher information measure-permutation entropy plane for all the daily discharge series analyzed and noise with power spectrum frequency-varying parameter *k* (gray dashed line). The parameters used are *D* = 5 and τ = 1.

**Figure 7:**
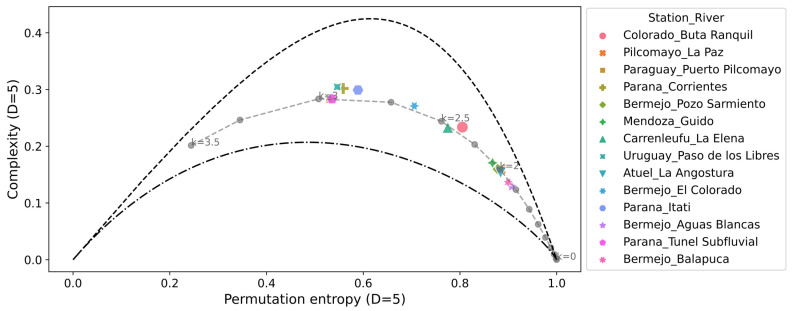
Complexity-entropy causality plane for all the daily deseasonalized discharge series analyzed and noise with power spectrum frequency-varying parameter *k* (gray dashed line). The parameters used are *D* = 5 and *τ* = 1.

**Figure 8:**
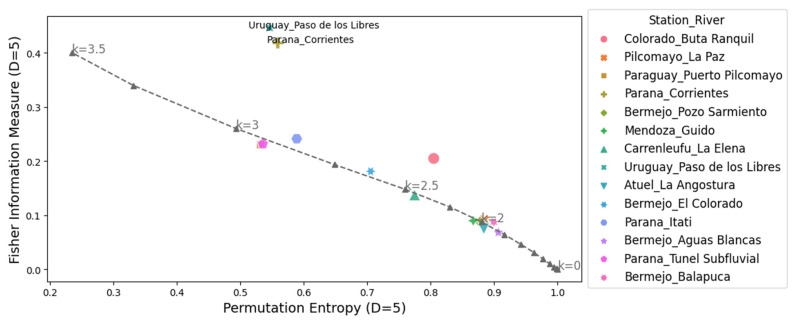
Fisher information measure-permutation entropy plane for all the daily deseasonalized discharges series analyzed and noise with power spectrum frequency-varying parameter *k* (gray dashed line). The parameters used are *D* = 5 and *τ* = 1.

The complexity-entropy causality plane (CECP) of Shannon and the Fischer–Shannon plane for the daily discharge series are shown in Figure 5 and Figure 6, respectively. The same procedures were repeated for the daily deseasonalized discharge series. They are shown in Figure 7 and Figure 8. The methodology was also applied to a dynamic stochastic series of k-noises (noise with power spectrum frequency dependence fitted by $f^{\left(-k\right)}$ values), with k ranging from 0.00 to 3.50, with intervals of 0.25 and 10 random simulations for every *k*.

The CECP showed that, in most cases, the daily streamflow series from different hydrometric stations exhibit similar behavior compared to Gaussian noise. In particular, this occurred for the parameter k between 2 and 3, and it was consistent with the results found in other studies [14,15]. The approximation to the noise series fits better in the deseasonalized discharge series, and the same conclusion is reached for the Fisher–Shannon plane. The results obtained from the Fisher–Shannon plane analysis for both the daily river discharge and its deseasonalized series. This revealed that the time series cannot be adequately described as Gaussian noise for the Paraná River at Corrientes station and the Uruguay River at Paso de los Libres station.

These findings demonstrated that modeling daily discharge series as Gaussian noise may be more appropriate when using deseasonalized series. However, the results highlight the difficulty of characterizing natural phenomena as either stochastic or chaotic. Not all discharge series exhibit the same behavior, requiring a thorough examination on a case-by-case basis. Quantifying the characteristics of different discharge series through information quantifiers allows for a better definition of which tools to employ in subsequent hydrological modeling, such as data-driven forecasting.

In [16,17], the authors investigated the influence of the construction of the Sobradinho dam on the daily streamflow of the São Francisco River. The authors found that there are different complexity and entropy patterns before and after the construction, in particular a higher permutation entropy. This shows that the operation of the reservoir induces decreased regularity. Following this methodology, a comparison of three selected stations was conducted. The generalized permutation entropy was calculated for the daily discharge time series at three stations downstream of the Yacyretá hydropower dam, situated in the upper basin of the Paraná River between Argentina and Paraguay. Yacyretá is a run-of-the-river power plant that has 20 Kaplan turbines with a total power of 3200 MW, and the area of the lake is 1600 km^2^ (Entidad Binacional Yacyretá). The comparison was conducted between the data series prior to the construction of the dam in 1983 and the period after 1994, when the first turbine became operational. The results are shown in Table 2.

Consistent with the findings in [16,17], the results showed that after the construction of Yacyretá, the permutation entropy was higher than before. This occurred even if the analysis was not conducted with data from a hydrometric station immediately downstream of the dam. The findings indicated that the operation of Yacyretá affects the randomness of the daily discharge series when the hydrometric station is immediately downstream of the dam. However, when the station is further downstream, this effect is attenuated.

The added value of this study lies in the conclusion that the impact of the dam operation on entropy diminishes as one moves further downstream from the dam. An explanation for that behavior is that the size of the basin plays an important role in modulating the process on a daily scale. Large catchments have smaller values for entropy, and the signal is less noisy due to integration over larger time scales [14].

Other studies have also found that the statistical properties of river flow fluctuations in daily data depend on the basin area [33,34]. For this reason, prediction models should not be directly transferred across watersheds of varying sizes without taking into consideration the effects of the basin area. The potential influence of a co-induced climatic effect, such as climate change or the El Niño/Southern Oscillation (ENSO), is not considered here. However, it could represent an additional factor contributing to changes in the dynamics of the discharge series.

These findings highlight that analyzing streamflow series entails examining the response to various processes occurring within the basin. Some are concentrated, others are spatially distributed, and their integration determines the varying degrees of influence among them. The information quantifiers could be used as a tool to assess the influence of each process and, therefore, use this knowledge to improve the modeling of water resources.

**Figure 9:**
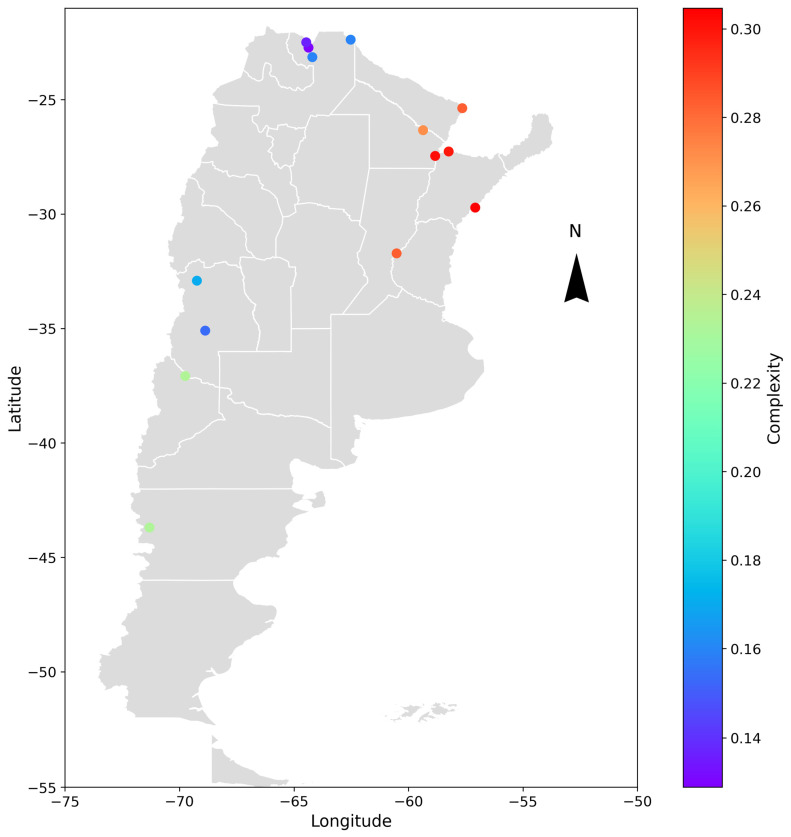
Geospatial representation of Argentina and the location of the analyzed hydrometric stations. Each point represents the varying degrees of complexity of the daily deseasonalized discharge series through a color-coded scale. The parameters used are *D* = 5 and *τ* = 1.

**Figure 10:**
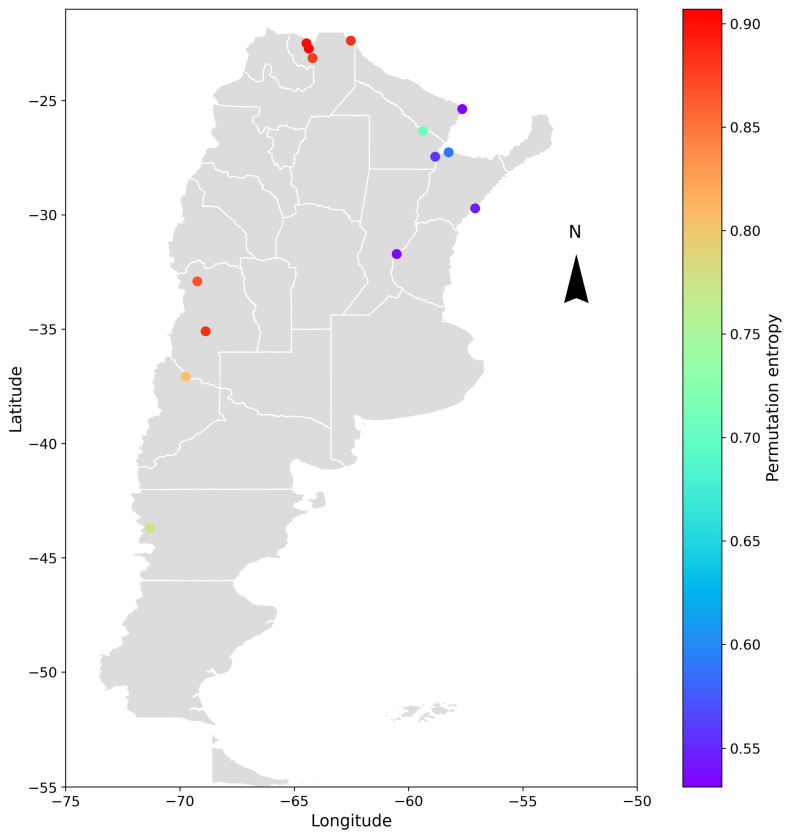
Geospatial representation of Argentina and the location of the analyzed hydrometric stations. Each point represents the varying degrees of permutation entropy of the daily deseasonalized discharge series through a color-coded scale. The parameters used are *D* = 5 and *τ* = 1.

**Figure 11:**
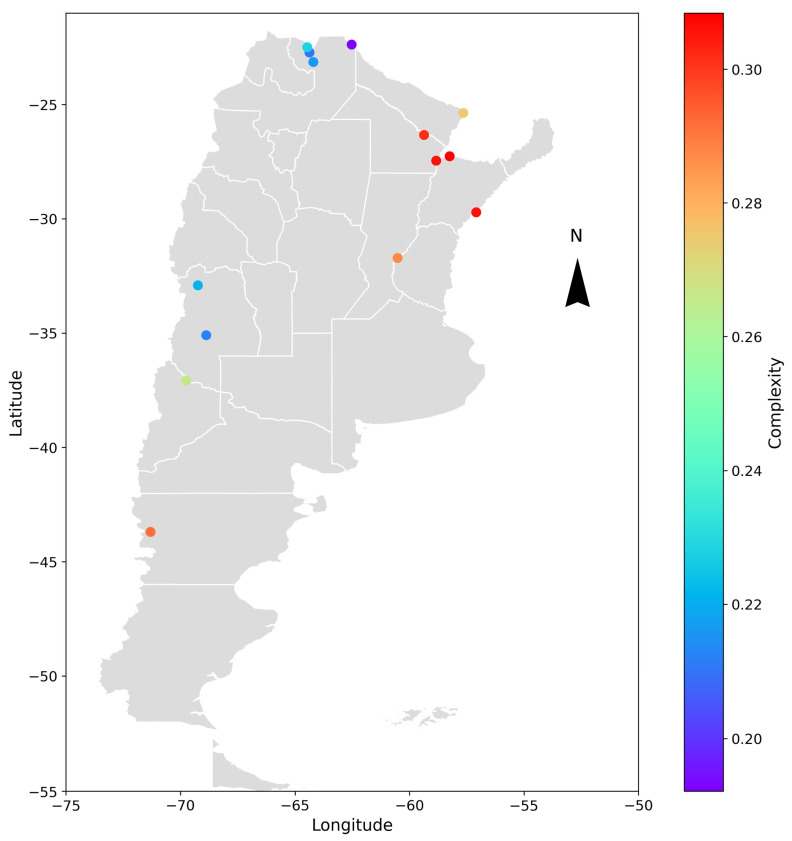
Geospatial representation of Argentina and the location of the analyzed hydrometric stations. Each point represents the varying degrees of complexity of the daily discharge series through a color-coded scale. The parameters used are *D* = 5 and *τ* = 1.

**Figure 12:**
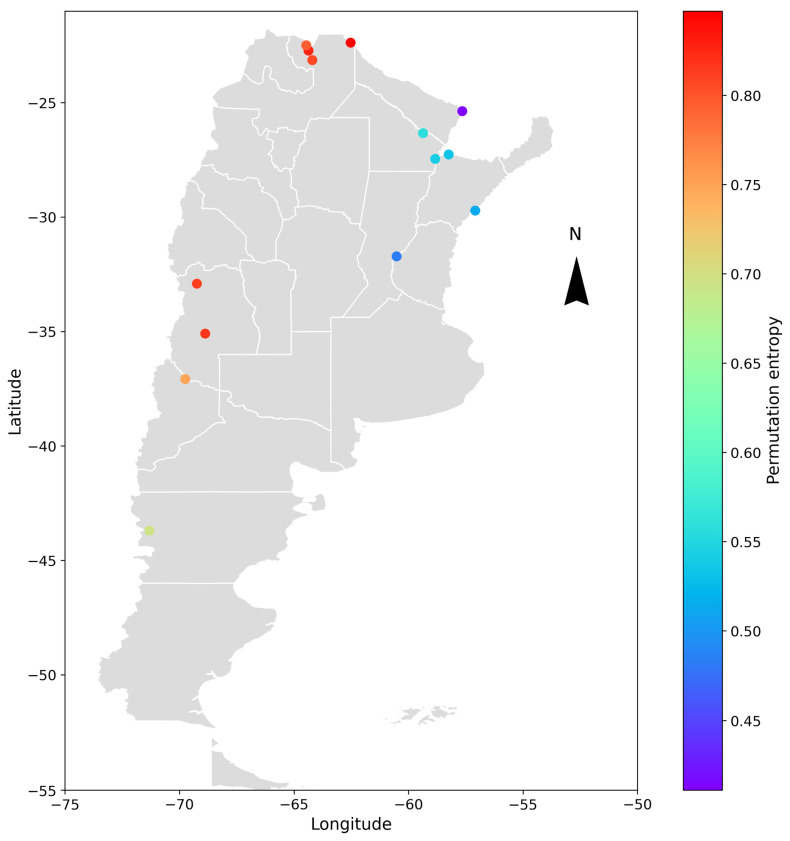
Geospatial representation of Argentina and the location of the analyzed hydrometric stations. Each point represents the varying degrees of permutation entropy of the daily discharge series through a color-coded scale. The parameters used are *D* = 5 and *τ* = 1.

A geospatial analysis was conducted to compare the different cases and determine if any patterns could be identified in the obtained results. The hydrometric stations were geographically located, and the entropy and complexity indicators were color-coded depending on the range of values obtained. The results are shown in Figure 9 and Figure 10 for the deseasonalized discharge series and in Figure 11 and Figure 12 for the daily discharge series.

On the one hand, Figure 9 and Figure 11 showed that the values for complexity of the discharge series seem to be similar to the deseasonalized discharge series. In both cases, the complexity varied approximately between 0.15 and 0.30 and was greater in the east than in the west, coinciding with the discharge series of less entropy.

On the other hand, Figure 10 and Figure 12 showed that for entropy, the results are similar for both the discharge series and the deseasonalized discharge series. In the first case, the entropy varied between 0.45 and 0.85. In the second case, it varied between 0.55 and 0.90, finding higher values in stations that are located in smaller basins in the west (except for the La Elena station) and decreasing values towards the east. The Paraná, Uruguay, and Paraguay rivers, along with the El Colorado station at the Bermejo River, exhibited the lowest entropy values among all stations.

The stations that presented higher entropy are La Angostura station in the Atuel River, Buta Ranquil station in the Colorado River, La Elena station in the Balapuca River, Aguas Blancas and Pozo Sarmiento stations in the Bermejo River, and Misión La Paz station in the Pilcomayo River. On the contrary, Corrientes and Túnel Subfluvial stations in the Paraná River, Paso de los Libres station in the Uruguay River, El Colorado station in the Bermejo River, and Puerto Pilcomayo station in the Paraguay River presented a lower value of entropy.

A possible explanation is that basins with a smaller area and the influence of highlands or mountains present a rapid response, affecting the behavior of the daily discharge. In basins with a larger area and flat topography, however, the response is slower. This implies that the catchment area plays a role in softening the daily variations and decreasing the degree of randomness in the series.

Based on these findings, it can be concluded that the geolocated representation of the results enables their integration with other influential features affecting discharge series, for example, topography and climate. This facilitates the unified analysis of results across diverse stations and offers the possibility of identifying patterns that can be further analyzed in future studies.

Nowadays, several hydrological modeling techniques are available for water resource forecasting and management. However, in countries like Argentina, where the distribution of water resources is heterogeneous and the characteristics of basins are diverse, selecting an accurate hydrological model can be challenging. The obtained results offer a framework for classifying different hydrological systems, facilitating more efficient model selection in order to support decision-making.

## 4. Conclusions

This paper analyzed the temporal evolution of the streamflow of different rivers in Argentina based on information quantifiers such as statistical complexity and permutation entropy. The main objective was to identify key details of the dynamics of the processes and quantify them to differentiate the degrees of randomness and chaos.

The analysis carried out for the shuffled series shows that the sorting of data has an impact on the structure and the measures of information.

The complexity-entropy causality plane (CECP) and the representation of the entropy and Fisher information measures showed that the daily discharge series could be approximately represented with Gaussian noise for a parameter *k* between 2 and 3. This approximation fits better for the deseasonalized discharge series. The Fisher–Shannon plane presented an important difference with respect to the Gaussian noise for the Corrientes station at the Parana River and Paso de los Libres at the Uruguay River, highlighting the difficulty of modeling a series of natural phenomena observed in real life.

An analysis of the daily discharge time series at the stations at the Paraná River downstream from the location where the Yacyretá hydroelectric dam was carried out for different periods. The results indicated that the operation of the dam impacts the randomness of the daily discharge series when the hydrometric station is in close proximity to the dam. However, this effect is attenuated when the station is situated further downstream. An explanation for such behavior may be that the size of the basin area plays an important role in modulating the process on a daily scale.

These findings highlight that analyzing streamflow time series entails examining the response to various processes occurring within the basin—some concentrated, others spatially distributed—whose integration determines the varying degrees of influence among them. The information quantifiers could be a tool to assess the influence of each process, thereby improving our ability to model water resources.

In addition, a geospatial analysis was carried out. On the one hand, small and mountainous basins presented a rapid response that influenced the behavior of daily discharge and consequently presented a higher entropy and lower complexity. On the other hand, basins with a larger area and smooth topography presented a slower response, and thus the results show a lower entropy and higher complexity. The catchment area plays a relevant role by softening the daily changes and decreasing the degree of randomness of the discharge series. The geolocated representation facilitated the unified analysis of results across diverse stations and offers the possibility of identifying patterns that can be further analyzed in future studies.

In this analysis, we found that information quantifiers such as permutation entropy and statistical complexity can assess the dynamics of daily discharge time series in Argentine rivers. In particular, the characterization of different basins and the impact of dam operation on the discharge series downstream were analyzed.

These findings highlight how information quantifiers can enhance our understanding of hydrological processes. This methodology not only provides a better insight into streamflow dynamics but also allows for the integration of this understanding into future hydrological models, enabling more accurate predictions and more effective water resource management strategies. Future research could involve investigating the influence of climatic effects such as climate change or the El Niño/Southern Oscillation (ENSO). Investigating the interconnections between these factors and their potential influence on hydrological patterns would provide a more holistic understanding of the evolving dynamics within water systems.

## Figures and Tables

**Table 1:** Basic information about the basins and records of discharge series used in this study.

River	Station	Basin Area (km^2^)	Record (Years)
Colorado	Buta Ranquil	15,300	1990–2019
Pilcomayo	La Paz	96,000	1960–2019
Paraguay	Puerto Pilcomayo	800,000	1910–2017
Paraná	Corrientes	1,950,000	1904–2019
Bermejo	Pozo Sarmiento	25,000	1940–2019
Mendoza	Guido	8180	1956–2019
Carrenleufú	La Elena	1500	1954–2019
Uruguay	Paso de los Libres	189,000	1908–2019
Atuel	La Angostura	3800	1931–2019
Bermejo	El Colorado	65,736	1968–2019
Paraná	Itatí	1,600,000	1910–2019
Bermejo	Aguas Blancas	4850	1944–2019
Paraná	Túnel Subfluvial	2,302,000	1904–2019
Bermejo	Balapuca	4420	1971–2019

**Table 2:** Comparison of the permutation entropy for the daily discharge series at Paraná River in three stations downstream of the Yacyretá dam before and after the construction.

River	Station	Basin Area (km^2^)	Permutation Entropy		
			Pre Yacyretá	Post Yacyretá	Variation
Paraná	Itatí	1,600,000	0.48	0.66	27%
	Corrientes	1,950,000	0.51	0.62	18%
	Túnel Subfluvial	2,302,000	0.47	0.50	6%

## Data Availability

The data used to support the findings of this study are included within the article. The processed data are available from the corresponding author upon request.

## Appendix A

In this appendix, the results obtained from applying the methodology to the daily discharge and deseasonalized discharge series are shown. The permutation entropy and statistical complexity were calculated for *τ* = 1 and varying *D* from 3 to 7 (Figure A1, Figure A2, Figure A3 and Figure A4).

The permutation entropy and complexity were applied to the daily discharge and deseasonalized discharge series for 3 < *D* < 7. In both cases, the permutation entropy decreases while the parameter *D* increases, and the results are shown in Figure A1 and Figure A2, respectively. For the complexity measure, as observed in Figure A3 and Figure A4, the behavior is different. The complexity and the parameter *D* increase simultaneously, but the slope is different among the time series studied. A possible explanation could be that the record period for the different series is not the same.

The same analysis was conducted for the shuffled series of daily discharge and the deseasonalized discharge series. The results show that in both cases, the entropy approaches 1 and the complexity approaches 0, as shown in Figure A5 and Figure A6 for daily discharge and Figure A7 and Figure A8 for deseasonalized discharge time series. These results indicate that the sorting of the values has an impact on the behavior of the time series in terms of entropy and complexity. These findings show that, for the studied discharge time series, the sequential arrangement of values significantly influences the process structure and the information obtained from it.
